# A Robust and Versatile Method of Combinatorial Chemical Synthesis of Gene Libraries *via* Hierarchical Assembly of Partially Randomized Modules

**DOI:** 10.1371/journal.pone.0136778

**Published:** 2015-09-10

**Authors:** Blagovesta Popova, Steffen Schubert, Ingo Bulla, Daniela Buchwald, Wilfried Kramer

**Affiliations:** 1 Department Molecular Microbiology and Genetics, Institute of Microbiology and Genetics, Georg-August-Universität Göttingen, Göttingen, Germany; 2 Department Molecular Genetics and Preparative Molecular Biology, Institute of Microbiology and Genetics, Georg-August-Universität Göttingen, Göttingen, Germany; 3 Department Dermatology, Venereology and Allergology, University Medical Center, Göttingen, Germany; 4 Information Network of Departments of Dermatology (IVDK), Göttingen, Germany; 5 Theoretical Biology and Biophysics, Group T-6, Los Alamos National Laboratory, Los Alamos, New Mexico, United States of America; 6 Institute for Mathematics and Informatics, Universität Greifswald, Greifswald, Germany; 7 Department Bioinformatics, Institute of Microbiology and Genetics, Georg-August-Universität Göttingen, Göttingen, Germany; 8 Neurobiology Laboratory, German Primate Center GmbH, Göttingen, Germany; 9 Department Molecular Genetics, Institute of Microbiology and Genetics, Georg-August-Universität Göttingen, Göttingen, Germany; Tel Aviv University, ISRAEL

## Abstract

A major challenge in gene library generation is to guarantee a large functional size and diversity that significantly increases the chances of selecting different functional protein variants. The use of trinucleotides mixtures for controlled randomization results in superior library diversity and offers the ability to specify the type and distribution of the amino acids at each position. Here we describe the generation of a high diversity gene library using tHisF of the hyperthermophile *Thermotoga maritima* as a scaffold. Combining various rational criteria with contingency, we targeted 26 selected codons of the *thisF* gene sequence for randomization at a controlled level. We have developed a novel method of creating full-length gene libraries by combinatorial assembly of smaller sub-libraries. Full-length libraries of high diversity can easily be assembled on demand from smaller and much less diverse sub-libraries, which circumvent the notoriously troublesome long-term archivation and repeated proliferation of high diversity ensembles of phages or plasmids. We developed a generally applicable software tool for sequence analysis of mutated gene sequences that provides efficient assistance for analysis of library diversity. Finally, practical utility of the library was demonstrated in principle by assessment of the conformational stability of library members and isolating protein variants with HisF activity from it. Our approach integrates a number of features of nucleic acids synthetic chemistry, biochemistry and molecular genetics to a coherent, flexible and robust method of combinatorial gene synthesis.

## Introduction

Proteins with novel and pre-determined properties, such as catalysis of chemical reactions or specific binding of low or high molecular weight ligands, are much sought for in biotechnology and biomedicine. Synthetic access to such proteins, however, is anything but straightforward, due to the fact that our knowledge of protein folding and structure/function relationship is still too fragmentary to allow deducing an amino acid sequence from nothing but the functional requirements it would have to meet.

For this reason, efforts to engineer proteins with new and pre-deliberated functions were originally confined to identifying functionally important single amino acid residues or small subsets of them within a pre-existing structural framework and replacing them by other residues of defined nature. This, by now classical, approach of directed mutagenesis (hypothesis-driven and "one molecule at a time") has more recently been complemented by methods sampling the sequence space using many candidate molecules in parallel and under incorporation of elements of chance, i.e. randomization of one or more amino acid positions. These methods are collectively known under the names of evolutionary and combinatorial protein engineering [1,2].

Repertoire diversity is a key parameter in such an approach because the probability of identifying one or several molecular species within a collection of partially randomized proteins as carriers of a specific predefined function increases linearly with the number of participating candidates. However, there are limits: With n fully randomized amino acid positions of a protein, the formal library size is 20^n^ or 10^1.3n^. For larger values of n, this number rapidly exceeds the number of molecules participating in a real life experiment. Under typical laboratory conditions the latter number is approximately as follows: 10^16^ for chemical oligonucleotide synthesis, 10^14^ for DNA ligation, 10^12^−10^14^ for ribosome display and 10^8^ for transformation of *E*. *coli* with plasmid DNA [3]. These numbers impose narrow constraints on what fraction of formal library diversity can actually be utilized in an experimental search for a given function.

Incentive is thus provided not to waste a large proportion of a gene library on candidates that have little if any chance to pass the functional test. This means that randomization ought to be directed away from residues involved solely in the maintenance of scaffold structure and stability and towards residues plausibly expected to contribute to the envisaged new function. In addition, it is highly attractive to also combine chance and beforehand knowledge by controlling not only the position but also the extent and quality of (partial) randomization.

This principle has paradigmatically been illustrated with libraries of immunoglobulins, or fragments thereof [4–8], guidance being provided by the general architecture of immunoglobulin variable domains. Here, structure-supporting residues are organized in "framework" regions of largely invariant sequence. These are interspersed by regions of great sequence flexibility which are present in the three-dimensional structure as clustered ensembles of "hypervariable loops" forming matching pockets for binding structurally diverse antigens [9,10]–hence the name "complementarity-determining regions" or "CDRs". With respect to enzymatic catalysis, a similar case is arguably provided by the (β/α)_8_-fold [11,12] which is highly prevalent among enzymes. By analogy, the regions corresponding to the CDRs of immunoglobulins are the eight loops connecting the C-termini of β-strands to the N-termini of α-helices. These invariably form the sites for substrate binding and catalysis. Utilization of this concept for finding (β/α)_8_-proteins with new and pre-determined catalytic properties requires sequence randomization addressing specifically those amino acid residues that are part of these loops and, in addition, extend their side chains towards the substrate binding cavity.

One of the methods to introduce random codons at specific positions within the synthetic DNA is oligonucleotide-directed mutagenesis (for reviews see [13,14]). In standard oligonucleotide-directed mutagenesis schemes, a randomized DNA sequence is synthesized by sequentially coupling a mixture of the four nucleoside precursors to the growing oligonucleotide. In this way all 64 possible codon sequences (NNN) are generated, including 41 redundant and 3 stop codons, where much of the clones obtained later are redundant or truncated. One way to improve this is to use NNS (S = G or C) or NNK (K = G or T). For both, there are only 32 possible codons with only one stop codon increasing the chance for productive clones, in particular if randomizing more than one position [15]. However, the bias in favor of the amino acids encoded by multiple codon sequences is maintained, and the presence of a stop codon will produce truncated amino acid sequences upon translation. This considerably limits the complexity that can be achieved for long randomized peptide libraries.

Several methods have been devised since to increase the quality of the library at the step of randomization. In the DimerTrimer method different pre-synthesized dimers and trimers are combined to yield only one codon per each of the 20 amino acid with no stop codons [16]. The same library quality can be obtained by the “small intelligent” focused mutagenesis [17]. Here, for each position to be randomized four oligonucleotides are needed, one with NDT (D = not C) representing 12 amino acids, one with VMA (V = not T, M = A or C) representing 6 amino acids and two with only one codon (ATG and TGG). Mixing the oligonucleotides in molar ratios of 12:6:1:1 should yield libraries without stop codons and an even distribution of each amino acid. It could be shown that using this method the library is considerably more balanced than using the NNS strategy. Recently, alternative methods for saturation mutagenesis have been described that include the “22c trick” [18], MAX and ProxiMAX randomization methodology [19,20]. These techniques use complex mixtures of oligonucleotides for saturation mutagenesis without bias, however the number of oligonucleotides required for randomization of more than 3 codons is impractical to handle. Of these only ProxiMAX, based on iterative cycles of blunt-ended ligation, PCR amplification and digestion, is suited to produce longer randomized contiguous codons, however with increased outlay of reagents and time.

A major drawback of all these methods is that there is no real control on the amount of randomization and the nature of the amino acid substitutions at any given position. A convincing solution of these problems is offered only by gene synthesis strategies, which incorporate trinucleotide phosphoramidites as coupling units, as has already been demonstrated for immunoglobulins [4–8]. Mixtures of such trinucleotide blocks are applied at every codon position to be fully or partially randomized. This achieves full position-wise control over the amino acid exchange probability and the composition of the participating set of residues. On the other side of the balance sheet are problems with synthesis and efficient coupling of trinucleotide blocks. Several syntheses of trinucleotide phosphoramidites are described in the literature [21–27] but there is still room for improvement. Despite the obvious attractiveness of trinucleotide phosphoramidites, their application is still limited to just a few published examples [4–8,28–33] among which the already mentioned randomization of immunoglobulin variable domains is the most prominent. Utilization of anticalins, non-immunoglobulin scaffolds with hypervariable loops, for the development of new therapeutic strategies is the most promising biomedical approach for protein engineering strategies [34].

Here we describe a novel method of combinatorial gene synthesis exemplified by tHisF, a thermostable (β/α)_8_-protein [35]. The following reasons prompted us to choose tHisF as a model: Many enzymes are endowed with only minimal folding stability in the sense that at physiological temperatures they operate close to conformational collapse [36]. As a thermostable protein, tHisF could be expected to provide a broader bandwidth of stability at 37°C making it possible to observe orderly folded variants with different degrees of conformational destabilization. The generation of the library is based on total gene synthesis in which mixtures of pre-synthesized trinucleotide phosphoramidites are employed for chain elongation whenever a codon site to be randomized is reached. The method combines the advantages of controlled codon randomization with hierarchical fragment combinatorics, based on stable and well-established laboratory procedures. The reliability of the method is critically assessed by sequence analysis of a larger number of candidate clones picked at random from libraries of genes and gene fragments. For efficient library analysis, we developed a freely available software tool (MUSI) for sequence analysis of mutated gene sequences. Finally, the quality of the library was tested by assessment of the conformational stability of library members and selection for protein variants with HisF functions.

## Materials and Methods

### Trimer phosphoramidite mixtures

Fully protected trimer phosphoramidites (protecting groups: 5'-O: Dimethoxytrityl; internucleotidic phosphate residues: 2-Chlorophenyl; 3'-O-Diisopropylphosphoramidite: 2-Cyanoethyl) were purchased from Glen Research, Sterling, VA. Trimer phosphoramidite mixtures were prepared as follows. A trimer representing a resident codon (0.7 equivalents) was mixed with a standard mixture of 19 trimers, representing all amino acids except cysteine (0.3 equivalents together). Ten such mixtures were required to serve all 26 codon positions to be randomized; they were purchased pre-mixed from the supplier. The standard mixture of 19 had the following composition (first number in brackets: molar fraction as intended to be realized at the protein level as amino acid residues; second number: correction factor applied by the supplier to amounts of respective trimers in order to compensate for empirically determined differences of incorporation rates in oligonucleotide synthesis). **A**: GCT (0.053/1.5), **D**: GAC (0.137/1.3), **E**: GAA (0.093/1.9), **F**: TTC (0.018/1.3), **G**: GGT (0.071/1.1), **H**: CAT (0.067/1.9), **I**: ATC (0.022/1.2), **K**: AAA (0.101/1.1), **L**: CTG (0.037/1.2), **M**: ATG (0.017/1.3), **N**: AAC (0.053/1.0), **P**: CCG (0.038/1.8), **Q**: CAG (0.046/2.0), **R**: CGT (0.081/1.1), **S**: TCT (0.052/1.3), **T**: ACT (0.048/1.3), **V**: GTT (0.028/1.9), **W**: TGG (0.007/2.4), **Y**: TAC (0.031/1.6). Cysteine was excluded from the mixture in order to avoid undesired disulfide bridges or oxidation reactions on the protein surface.

### Oligonucleotides

Oligonucleotides were chemically synthesized by PURIMEX, Grebenstein, Germany, at 100 nmol scale using an ABI model 392/394 synthesizer and applying standard conditions with the following modifications. Support: 2000 Å controlled pore size glass (ChemGenes, Wilmington, MA). Coupling catalyst: 0.2 M 5-Benzylmercaptotetrazole (emp Biotech, Berlin, Germany). Coupling time: 24 sec (monomers), 5 min (trimers). Base and phosphate deprotection: 32% ammonia, room temperature, 17 h, followed by 55°C, 4 h. Products were purified by two rounds of HPLC (5'-dimethoxytrityl groups on/off; matrix: Oligo R3, Perseptive Biosystems, Framingham, MA), followed by preparative PAGE. Yields ranged from 3 to 5 nmol.

Sequences of chemically synthesized oligonucleotides (T1—T13) are listed below. Following the name of each oligonucleotide, its number of residues and the restriction sites flanking the corresponding double-stranded gene module are given in parentheses. Randomized positions are underscored with indicated sequences representing the corresponding wild type codons. Oligonucleotide T5 (two randomized positions) was used in the synthesis of library L26; for synthesis of L24, a second version of T5 was prepared with wild-type sequence throughout. The term "wild type" is used in the sense of "coding for wild-type protein sequence (including the C9A exchange)".

#### T1 (73, Smil, Bsal/ Esp3I)

ATTTAAATGGTCTCCAATGGTCGCTAAACGTATAATCGCTGCTCTCGACGTGAAGGATGGTCGTGTGGAGACG

#### T3 (67, Esp3I/ BsaI, SmiI)

CGTCTCCCGTATTTCTGGATATCACTGCTTCTGTTGAGAAGCGTAAGACTATGGAGACCATTTAAAT

#### T4 (57, Smil, Bsal/ Esp3I)

ATTTAAATGGTCTCCCTATGCTGGAACTGGTTGAGAAGGTGGCCGAGCAGGGAGACG

#### T5 (86, BsaI/ BsaI)

GGTCTCCGCAGATTGATATTCCGTTCACTGTT(GGT)GGTGGTATC(CAT)GACTTTGAGACGGCCTCTGAACTGATTCGGAGACC

#### T6 (82, Esp3I/ BsaI, SmiI)

CGTCTCCATTCTGCGTGGTGCTGACAAGGTGTCTATTAACACTGCTGCTGTGGAAAATCCTTCTCTGGGAGACCATTTAAAT

#### T7 (70, Smil, Bsal/ Esp3I)

ATTTAAATGGTCTCCTCTGATTACACAGATCGCTCAAACCTTCGGGAGTCAGGCTGTTGTTGTGGAGACG

#### T8 (82, Esp3I/ Esp3I)

CGTCTCCttgtgGCTATAGACGCTAAGCGTGTGGATGGAGAGTTTATGGTATTCACCTACTCTGGTAAGAAGAACGGAGACG

#### T9 (82, Esp3I/ Esp3I)

CGTCTCCGAACACGGGTATCCTGCTTCGTGACTGGGTGGTTGAAGTAGAGAAGCGTGGAGCAGGAGAGATTCTTCGGAGACG

#### T10 (69, Esp3I/ Bsal, Smil)

CGTCTCCCTTCTTACTAGTATCGACCGTGACGGTACAAAATCGGGTTATGATAGGAGACCTATTTAAAT

#### T11 (76, Smil, Bsal/ Esp3I)

ATTTAAATGGTCTCCGATACTGAGATGATTCGTTTCGTGCGTCCACTGACCACACTTCCGATCATTGCTGGAGACG

#### T12 (64, Esp3I/ Esp3I)

CGTCTCCTGCTTCTGGTGGTGCTGGTAAGATGGAACATTTCCTTGAGGCATTTCTGGGGAGACG

#### T13 (62, Esp3I/ Esp3I)

CGTCTCCCTGGCAGGTGCTGATGCTGCGCTGGCTGCTTCTGTGTTCCACTTTCGTGGAGACG

Fragments C2 and C14 (no randomized positions) were amplified by PCR from a previously synthesized *thisF* (C9A) gene (this laboratory, unpublished). The following primer pairs were used:

**C2-for.** (CGTCTCCGTGTGGTGAAGGGCACTAAC);

**C2-rev.** (CGTCTCCTACGA-GTTCATCTATACCAAT);

**C14-for.** (CGTCTCCTCGTGAGATTGATGTTCG);

**C14-rev.** (ATTTAAATGGTCTCTGCTATCTAGTGG).

The sequence of the PCR products is as follows:

#### C2 (112, Esp3I / Esp3I)

CGTCTCCGTGTGGTGAAGGGCACTAACTTTGAGAACCTGCGTGACAGCGGCGATCCTGTGGAACTGGGTAAATTCTACTCTGAGATTGGTATAGATGAACTCGTAGGAGACG

#### C14 (121, Esp3I / BsaI, SmiI)

CGTCTCCTCGTGAGATTGATGTTCGTGAACTGAAAGAGTATCTGAAGAAGCACGGAGTGAATGTACGTCTGGAGGGTTTGCACCACCACCACCACCACTAGATAGCAGAGACCATTTAAAT

### Klenow fill-in reaction

The oligonucleotides T1-T13 were converted to dsDNA fragments by Klenow fill-in reaction using primers:

**T1-rev.** (CGTCTCCACACGACCATCCT);

**T3-rev**. (ATTTAAATGGTCTCCATAGTCTTAC);

**T4-rev.**(CGTCTCCCTGCTCGGCCAC);

**T5-rev**. (GGTCTCCGAATCAGTTCAG-AG);

**T6-rev.** (ATTTAAATGGTCTCCCAGAGAAGGAT);

**T7-rev.** (CGTCTCCACAACAACAGCCT);

**T8-rev.** (CGTCTCCGTTCTTCTTACC);

**T9-rev.** (CGTCTCCGAAGAATCTCTCCT);

**T10-rev.** (ATTTAAATAGGTCTCCTATCATAACCC);

**T11-rev.** (CGTCTCCAGCAATGATCGGAAGTG);

**T12-rev.** (CGTCTCCCCAGAAATGCCTC);

**T13-rev.** (CGTCTCCACGAAAGTGGAACAC)

100 pmol oligonucleotide template, 150 pmol primer, 20 nmol dNTP mix and 5μl 10x reaction buffer for Klenow Fragment were mixed in total volume of 50 μl. The mixture was incubated at 80°C for 2 min. followed by 1 min. primer annealing at 50°C. The fill-in reaction was incubated for 10 min. at 37°C after addition of 10 U Klenow fragment. The DNA was ethanol precipitated and used for further cloning.

### Construction of plasmid fragment libraries

The Klenow fill-in products were cloned in Zero Blunt TOPO Vector (Invitrogen) or in pJET1.2 (Thermo Scientific) cloning vector (C-fragment libraries). The transformation reactions of the cloned randomized fragments were plated on 2 large plates each (d = 14.5 cm) containing LB-medium supplemented with corresponding antibiotics (TOPO: 75μg/ml kanamycin; pJET1.2: 100 μg/ml ampicillin). For determination of the size of the fragment libraries, a portion of the transformation reaction was plated on a small plate (d = 8.5 cm). The transformants grown on the large plates were scraped off under sterile conditions with liquid dYT medium and the resulting cell suspensions were used for preparation of randomized plasmid libraries. The same procedure was used for the generation of plasmid encoded B-fragment libraries.

### Exonuclease V treatment

All plasmid preparations were treated with ExonucleaseV (USB Corporation, Cleveland), which hydrolyzes nucleotides from both the 3' and 5' ends of linear double-stranded DNA. Typically 150 μg plasmid DNA were incubated for 30 min. at 37°C with 5 U of Exonuclease V in assay buffer containing: 66.7 mM glycine-NaOH buffer (pH 9.4), 30 mM MgCl_2_, 8.3 mM 2-mercaptoethanol, 0.5 mM ATP in a final volume of 300 μl. The enzyme was heat-inactivated for 10 min. at 65°C and the DNA desalted with Wizard SV Gel and PCR Clean-Up System (Promega).

### Restriction digestion and ligation assembly

The plasmid-encoded C-fragment libraries were subjected to restriction digestion using the restriction endonucleases SmiI, Esp3I or BsaI. In the case of end fragments, the plasmid libraries were linearized with SmiI, dephosphorylated with CIAP and after inactivation of the alkaline phosphatase a second restriction digestion with Esp3I was done. The C-fragment libraries coding for internal fragments were digested with Esp3I (except P5, where BsaI was used). The restriction fragments were gel-eluted from PAGE (12% PAA) or from agarose gels with QIAEX II Gel Extraction Kit (Qiagen) and their concentration was determined spectrophotometrically. Stoichiometric amounts of the fragments, participating in one ligation assembly were mixed and the ligations were incubated overnight at 16°C using T4 DNA Ligase. The full-length ligation products were gel-eluted using Wizard SV Gel and PCR Clean-Up System (Promega) and used for further cloning into Zero Blunt TOPO Vector (Invitrogen) or in pJET1.2 (Thermo Scientific) cloning vector for generation of B-fragment libraries.

### Construction of full-length gene plasmid library

All B-fragment libraries were subjected to restriction digestion with BsaI and the restriction fragments–gel-eluted with Wizard SV Gel and PCR Clean-Up System (Promega). The gene ligation assembly was carried out as described above and the full-length assembly product was gel-eluted. For production of soluble tHisF variants, the library was directionally cloned into the expression vector pASK-08 *via* two BsaI restriction sites. The vector pASK-08 is a derivative of the commercially available pASK-IBA3 (IBA GmbH, Germany). The 4-base 5’ sticky end of the downstream BsaI cloning site was replaced by quick-change mutagenesis (QuikChange Site-Directed Mutagenesis Kit, Stratagene) from the palindromic GCGC to TAGC using the primers: **pASK-mut-for**: CCATGGTCTCATAGCTTGGAGCCAC and **pASK-mut-rev**: GTGGCTCCAAGCTATGAGACCATGG.

Vector pASK-08 was treated with ExoV for elimination of possible *E*.*coli* genomic contaminations (see above) and after restriction digestion with BsaI the vector was purified by a sucrose gradient centrifugation. Ten ligation reactions were prepared, each containing 200 ng vector and 2 fold molar excess of insert DNA, 2 μl T4 DNA Ligase, 2 μl 10x T4 DNA Ligase buffer in a final volume of 20 μl over night at 16°C. The ligations were pooled, extracted once with phenol/chloroform, the DNA was precipitated and used for ten transformations of DH5α *E*.*coli* strain *via* electroporation. One ml SOC medium was added to each electroporation mixture and after incubation for 1 h at 37°C on a roller, the cells were pooled and plated onto 40 big LB petri dishes (d = 14.5 cm). The bacteria were recovered from the plates by scraping them under sterile conditions with liquid dYT medium and the resulting cell suspension was used for preparation of the plasmid library without additional growth.

### DNA Sequence analysis

DNA sequencing was done at Helmholtz Centre for Infection Research, Braunschweig, Germany. The DNA was isolated in 96-well Millipore plates (MAFB N0B 50, MANANLY 50). The sequencing was done on ABI 3730xl DNA Analyzer with BigDye Terminator v3.1 Cycle Sequencing Kit using the following sequencing primers:

pASK-for.2 (for sequencing in forward orientation): GAGAAAAGTGAAATGAATAGTTCG

pASK-rev.2 (for sequencing in reverse orientation):

TTCACTTCACAGGTCAAGC.

### MUSI (Mutation sites) sequence analysis tool

The MUSI software tool was designed for sequence analysis of mutated gene sequences both by site-directed mutagenesis and by standard methods of random mutagenesis like error-prone PCR. MUSI is freely available for download at http://bioinf.uni-greifswald.de/bioinf/musi/musi.html. The tool identifies the mutations and exports them in Excel data sheets. The tool has to be provided with a reference sequence (the scaffold sequence) and the sequences to be compared to the reference sequence (the sequences obtained by mutating the scaffold sequence). It then fulfills the task of generating a detailed report on mutation events on nucleotide, codon, and amino acid level as well as summary analysis of the mutation events. The reference sequence, the target sequences, and—in case of site-directed mutagenesis—a sequence indicating the codon positions targeted by the directed mutagenesis have to be provided in FASTA format in aligned form (any standard multiple sequence alignment software will work for this). Hereby, the number of target sequences is limited to 250. MUSI is implemented in Java and runs on Windows as well as UNIX based systems. It has not been tested for Mac OS. Further details about how to use MUSI are provided in the manual that is included in the download package.

#### Output of MUSI

The output analysis conducted by MUSI is organized as tables in five separate Excel sheets. In detail, these sheets contain the following information:

1. Nucleotide substitutions and deletions of nucleotides occurring in the target sequences on a single nucleotide level, stratified by target sequence. This analysis does not incorporate the positions of the codons targeted by directed mutagenesis but treats all codons equally;
2. Nucleotide substitutions and deletions of nucleotides occurring in the target sequences on a codon level, stratified by target sequence. Depending on whether site-directed or random mutagenesis is considered, either substitutions at the targeted codons are displayed or at all codons;
3. Randomized codons are translated into amino acid residues and are listed at the corresponding position for each individual sequence. Depending on whether site-directed or random mutagenesis is considered, either substitutions at the targeted codons are displayed or at all codons. Moreover, summary statistics for deletion events is provided for each target sequence;
4. Amino acid substitutions occurring in the target sequences, stratified by the type of the replacing amino acid; total number of substitutions per position and per type of residue; total number of single, double or triple nucleotide deletions;
5. Number and type of transitions, transversions and nucleotide deletions outside of the randomized codons along the whole DNA sequence.

#### Running time

Analysis of 43 target sequences with 26 codons, mutated by directed mutagenesis, with alignment length of 1702 nucleotides, results in a running time of about 3 seconds on a Dell Latitude D520 with a Intel Core Duo T2300 1.67 GHz for generating all five output tables.

### Analysis of protein solubility

In order to determine the fraction of soluble protein variants in the library, single random clones were picked from non-selective plates and examined for the presence of soluble tHisF variants. Single clones were grown at 37°C in dYT culture supplemented with 100 μg/ml ampicillin. Overexpression was induced by addition of anhydrotetracycline to a final concentration of 200 ng/ml at an optical density of 0.6 at 600 nm and incubation was continued for 3 hours. The cells were harvested by centrifugation and lysed by sonification (30 s) in buffer, containing 100 mM Tris pH 8, 150 mM NaCl. The crude cell extract was centrifuged for 30 min. at 13000 rpm at 4°C to separate the soluble from the insoluble fraction. Cell extract concentrations of the supernatants were determined by a standard Bradford assay [37]. Equal amounts of total protein were applied on a SDS-PAGE and Western blot was performed under standard conditions with Anti-His(6)-antibody.

### Genetic complementation for selection of protein variants with HisF activity

The plasmid library preparation was used for electroporation of competent auxotrophic *E*.*coli* ΔhisF cells (Keio collection, National BioResource Project (NIG, Japan, JW2007). After one hour recovery in SOC medium at 37°C, the transformants were washed 2 times with PBS buffer and streaked on large M9 minimal plates, containing 100 μg/ml ampicillin, 200 ng/ml anhydrotetracycline and amino acid mix 19 (all amino acids except histidine—40 μg/ml; Trp– 20 μg/ml). As a negative control, cells were transformed with empty pASK-08 vector and as a positive control–with pASK-08-tHisF (wild type). The plates were incubated at 37°C for 2 days. At that time, visible colonies have grown both on selection plates and on those transformed with the positive control. In contrast, no growth could be detected on the negative control plates, transformed with the empty vector, even by prolonged incubation (5 days). A total of 287 clones were counted on the selective plates by a total titer of library transformants on non-selective LB-plates, containing 100 μg/ml ampicillin, of 1.8 x 10^6^. Single clones were picked from the selective plates and plasmid preparations were done. The plasmid preparations were used for single retransformations in *ΔhisF* cells for confirmation of the genetic complementation and the DNA sequence of the clones was determined.

### Growth curves

Several selected library members that complemented the hisF deficiency most efficiently were further investigated to determine the growth rate of *ΔhisF* cells, transformed with the corresponding plasmid variants. The transformants were used to inoculate 50 ml of M9 minimal medium, containing 100 μg/ml ampicillin, 75 μg/ml kanamycin, 200 ng/ml anhydrotetracycline, 50 mM (NH_4_)_2_SO_4_ and amino acid mix with all amino acids except histidine at a concentration of 4 mg/ml each. Positive and negative controls were grown in parallel. Over night cultures were diluted to OD_600_ = 0.1 and the optical density was measures every 45 min. The optical density was plotted against time and the doubling time was determined. The data are the mean of at least 5 independent experiments.

## Results and Discussion

### Library design

#### Choice of the model protein and identification of favorable residues for randomization

**Fig 1:**
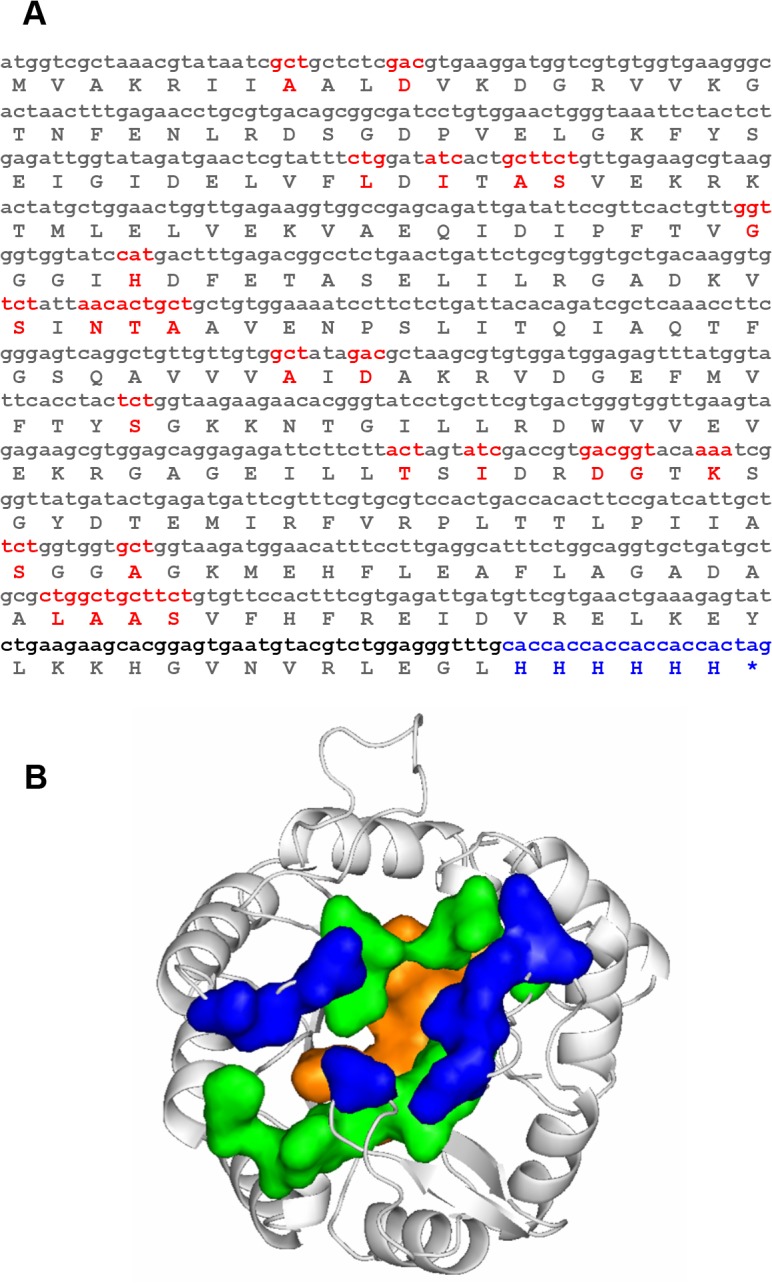
Amino acid sequence of tHisF and randomized positions. (A) Nucleotide sequence of synthetic *thisF* gene. The protein comprises 253 amino acid residues, corresponding to 759 nucleotide of its structural gene. His6-tag was fused C-terminally to the coding sequence (depicted in blue together with stop codon). Red font indicates randomized positions. (B) Three dimensional location of randomized chain positions. The residues selected for randomization are presented as surfaces and colored according to their distance from the top (C-terminal ends of the β-strands). Blue: I52, A54, S55, S144, I173, D176, G177, K179; green: D11, L50, G80, H84, N103, T104, A105, D130, T171, S201, A204, A224, S225: orange: A8, S101, A128, L222, A223.

As a model case for the approach outlined in the Introduction, HisF (imidazole glycerol phosphate synthase) of the hyperthermophile *Thermotoga maritima* (“tHisF”) [35] was chosen (see Fig 1A). Residues to be randomized were selected according to the following criteria: *(i)* Selected residues line the substrate-binding cleft. *(ii)* Their side chains are oriented towards the barrel axis. Gly 81 and Gly 82, though meeting the above criteria, were not included in the set because they have combinations of dihedral angles Φ and Ψ not commonly observed with other amino acids. A total of 26 residues were identified, all of which are clustered near the borders between C-termini of β-strands and N-termini of β/α loops (see Fig 1B).

#### Randomization parameters

**Fig 2:**
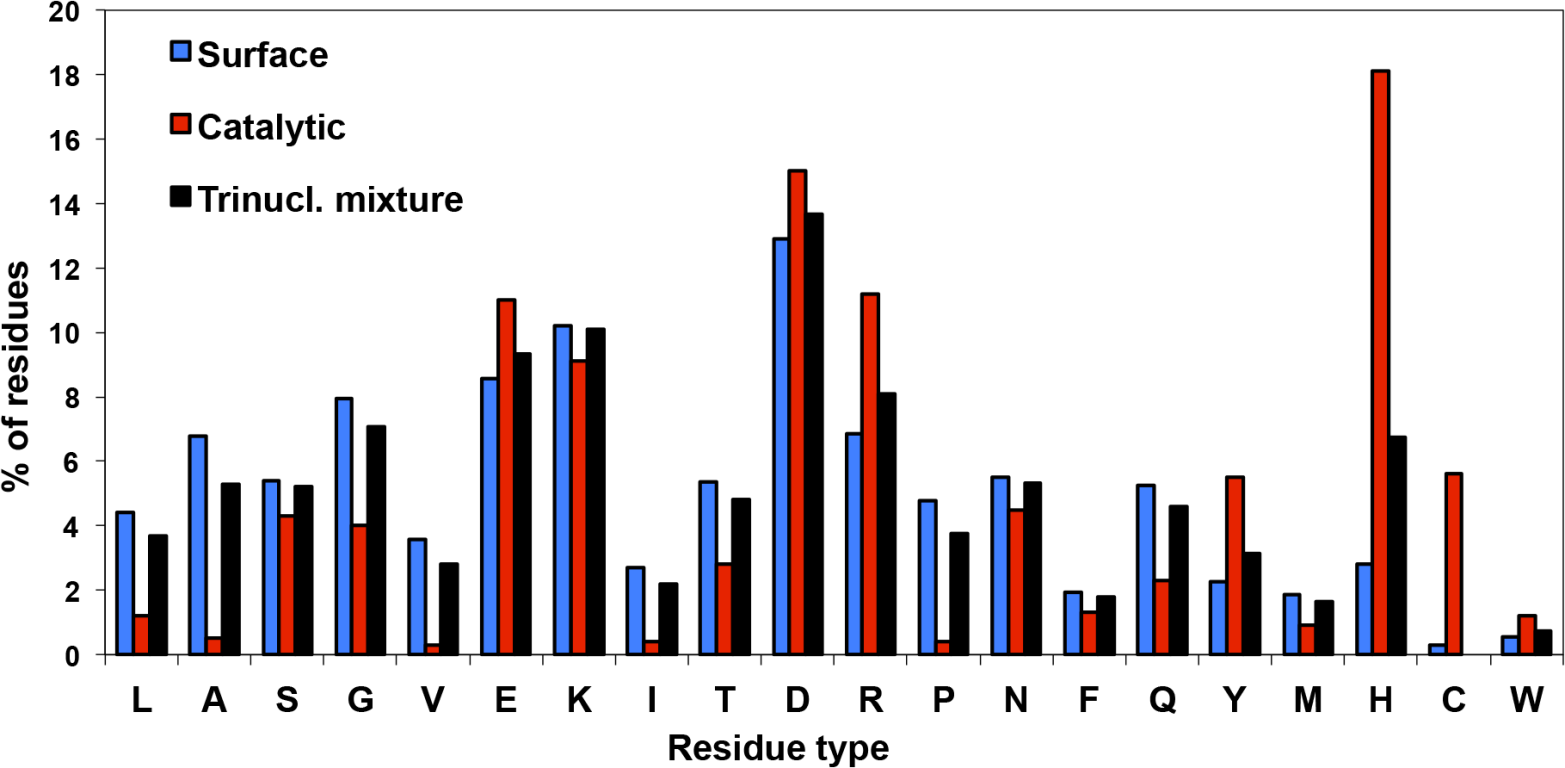
Molar fractions of 19 codons in standard mixture and their derivation from observed occurrences on the surface of proteins and in catalytic sites of enzymes. The frequency of the residues in the trinucleotide mixture is calculated from the surface residue frequencies (mesophiles) [38], multiplied with a factor of 0.75, plus catalytic residue frequencies [39], multiplied with a factor of 0.25. The frequency for cystein was set to zero and all other values were normalized to 1.

Parameters for incorporation of mixtures of trinucleotides were set as follows and applied in identical fashion to all randomized positions: *(i)* A standard mixture of 19 trinucleotide phosphoramidites was prepared representing all amino acids except cysteine. The latter was excluded in order to avoid complications arising from oxidation at the protein level. *(ii)* For each position, the trinucleotide representing the wild type residue was added to the standard trinucleotides mixture at a molar fraction of 0.7. Since the respective wild type trinucleotides were also present in the standard mixture–and to a variable degree (see below)–this lead to exchange probabilities per codon position closely clustered around an average of 0.28. For a library with 26 randomized positions this results in expected 7.31 codon exchanges on average; for 24 randomized positions in 6.69 (see below). *(iii)* A bias was introduced into the spectrum of exchange propensities of individual amino acids in favour of known surface [38] and catalytic [39] residues as follows. Molar fractions of corresponding trinucleotides in the standard mixture were adjusted to weighted means of their observed occurrence with weighting factor 0.75 for surface residues and 0.25 for catalytic residues (see Fig 2). Calculated molar fractions were further corrected for different chain elongation kinetics; for details refer to Materials and Methods.

### Library synthesis

#### Synthesis and assembly strategy

**Fig 3:**
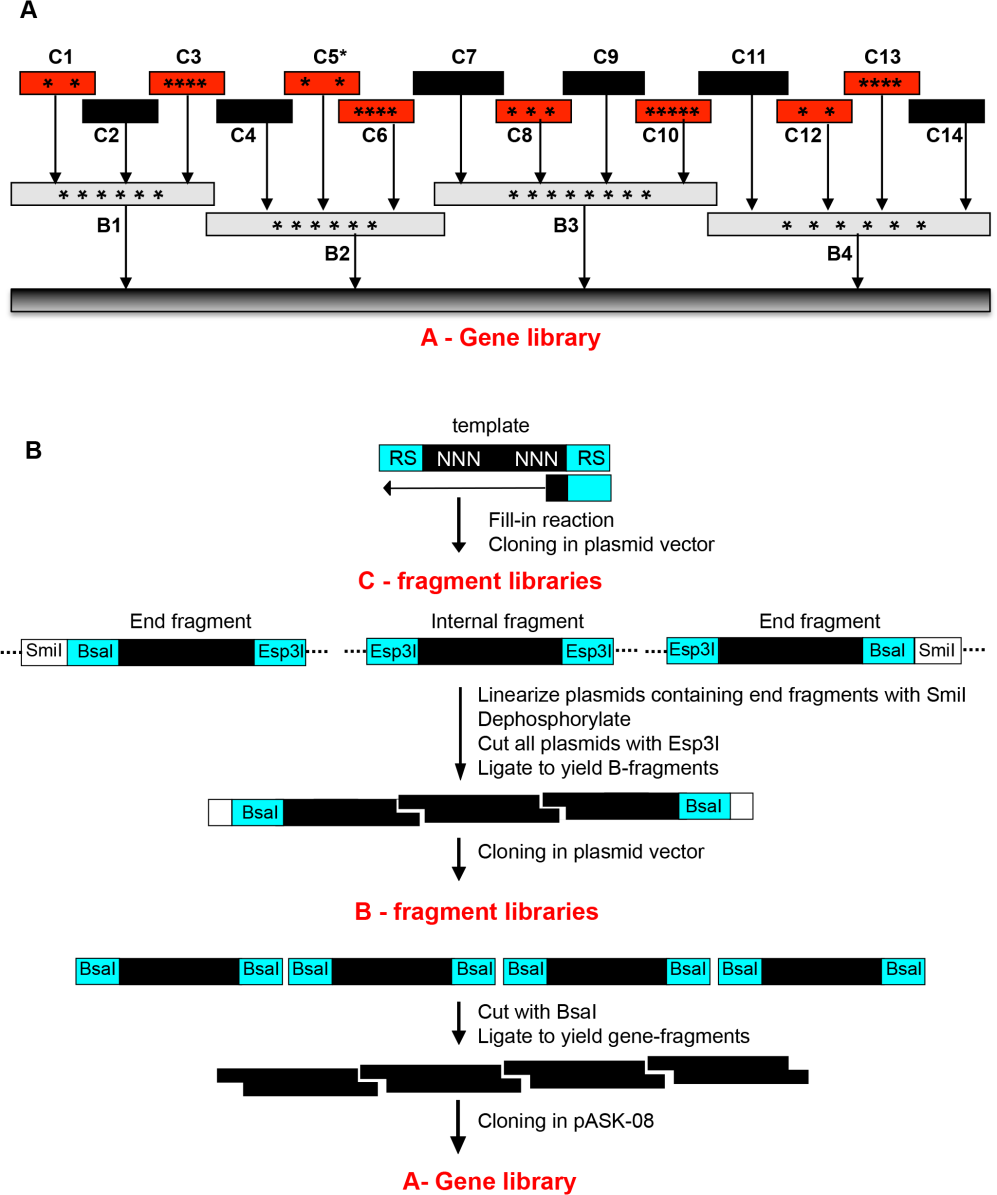
Modular division of *thisF* gene and hierarchical assembly scheme. (A) The gene sequence was divided into 14 modules (C1-C14). The black bars represent modules with wild type sequence; the red bars represent randomized sequences. The modules were chemically synthesized and randomization, which was achieved by incorporating trinucleotide mixtures at the corresponding codon positions, indicated by asterisks. Module C5 was synthesized both as wild type and as randomized sequence. The gene library was generated by combinatorial assembly of smaller modules (fragment libraries) at two steps, schematically represented with arrows. (B) Enzymatic steps involved in generation of double-stranded fragments and step-wise gene assembly. All C-fragments were designed in such a way that recognition sequences of Type RII restriction enzymes are located outside the coding sequence and are removed in the act of cleavage, whereas cleavage points in both DNA strands are within coding sequence but outside randomized DNA sites. Also refer to Table 1.

The *thisF* gene to be synthesized as a library was broken down into 14 DNA modules to be assembled in two hierarchical steps of DNA ligation as illustrated in Fig 3A. The various biochemical reactions employed for gene assembly are indicated in Fig 3B. PCR was avoided in all steps involving randomized DNA in order to safeguard against possible amplification bias. Two longer fragments (C2 and C14), containing no randomized positions, were amplified by PCR. At the core of the strategy are single round DNA polymerase reactions and the potential of certain "Type IIS" restriction enzymes (in this case Esp31 and BsaI) to generate protruding DNA ends of arbitrarily chosen sequence [40] (compare Table 1). This allows splitting of the gene into modules at any nucleotide outside of the randomized positions.

**Table 1:** Positions of fragment borders within synthetic genes L26 and L24.

Fragment	Upstream protrusion		Downstream protrusion	
	Position	Sequence upper strand, 5'→3'	Position	Sequence lower strand, 5'→3'
C1	– 1 to 3	AATG	50 to 47	ACAC
C2	47 to 50	GTGT	144 to 141	TACG
C3	141 to 144	CGTA	185 to 182	ATAG
C4	182 to 185	CTAT	216 to 213	CTGC
C5	213 to 216	GCAG	280 to 277	GAAT
C6	277 to 280	ATTC	336 to 333	CAGA
C7	333 to 336	TCTG	380 to 377	ACAA
C8	377 to 380	TTGT	444 to 441	GTTC
C9	441 to 444	GAAC	508 to 505	GAAG
C10	505 to 508	CTTC	550 to 547	TATC
C11	547 to 550	GATA	600 to 597	AGCA
C12	597 to 600	TGCT	646 to 643	CCAG
C13	643 to 646	CTGG	690 to 687	ACGA
C14	687 to 690	TCGT	+1 to 778	GCTA

Non-symmetrical and uniquely pairwise complementary 5'-protrusions created at ends of DNA fragments C1 to C14 by Type RII restriction enzymes Esp31 and BsaI (compare Fig 3).

Eight synthetic modules (marked red in Fig 3A) contain between two and five randomized positions. At the lowest hierarchical level ("C"), this results in fragment libraries of low to moderate diversity (361 to 2.5x10^6^, compare Table 2), which facilitates their archival as plasmid libraries without loss of diversity.

**Table 2:** Formal *vs*. experimental diversity.

Fragment library	Theoretical diversity	Number of independent clones
**C1 (2)**	3.6 x 10^2^	6.9 x 10^3^
**C3 (4)**	1.3 x 10^5^	2.4 x 10^3^
**C5 (2)**	3.6 x 10^2^	2.6 x 10^3^
**C6 (4)**	1.3 x 10^5^	1.0 x 10^3^
**C8 (3)**	6.9 x 10^3^	2.9 x 10^3^
**C10 (5)**	2.5 x 10^6^	1.8 x 10^3^
**C12 (2)**	3.6 x 10^2^	4.9 x 10^3^
**C13 (4)**	1.3 x 10^5^	9.0 x 10^3^
**B1 (6)**	4.7 x 10^7^	3.0 x 10^4^
**B2 (6)**	4.7 x 10^7^	1.0 x 10^4^
B2*(4)	1.3 x 10^5^	3.7 x 10^4^
**B3 (8)**	1.7 x 10^10^	1.6 x 10^4^
**B4 (6)**	4.7 x 10^7^	3.4 x 10^4^

The number of randomized positions in each fragment library is indicated in brackets. The theoretical diversity represents the number of all possible combinations of 19 different codons by the given number of randomized positions. Fragment library B2(6) was used for the synthesis of L26; fragment library B2*(4)–for the synthesis of L24.

#### Generation of level C-fragments and C-fragment libraries

Twelve synthetic C-fragments have lengths between 57 and 86 nucleotides which is well within reach of chemical DNA synthesis. These were converted to blunt-end double stranded DNA by fill-in reaction using the Klenow fragment of DNA polymerase I (Fig 3B, top). Two modules not containing any randomized positions (C2 and C14 with lengths of 112 and 121 nucleotides, respectively) were prepared by PCR-copying from a previously synthesized *thisF* gene encoding wild type tHisF (this laboratory, unpublished).

The resulting blunt-end fragments were analyzed by PAGE (S1 Fig) and inserted into a TOPO vector, generating C-fragment clones (“black fragments"–compare Fig 3A) and C-fragment libraries (“red fragments"). Transformation efficiencies of the eight C-fragment libraries were in the order of several thousand each (Table 2). According to our experience, such small numbers are not uncommon in transformation experiments involving chemically synthesized DNA. Thus, multiplicity of coverage of the theoretical combinatorial diversity spans a spectrum of approximately 19 fold (C1) to 10^−3^ fold (C10). Due to two additional stages of combinatorial mixing (fragment ligation to yield libraries at levels B and A), as few as one thousand different sequences present in each of the eight C-module libraries would still open up a sequence space, available at level A, of (10^3^)^8^ = 10^24^ distinct molecular species–a merely formal number, way beyond the number of DNA molecules handled at any time during this experiment or restricted by the transformation bottlenecks.

One clone of confirmed sequence was kept for further use from each non-randomized ("black") fragment; "red" fragments were stored as plasmid libraries. From each of these, a larger number of unselected clones was sequenced and statistically analyzed as illustrated in detail under "library analysis" (see below).

#### Generation of B-fragment libraries

**Fig 4:**
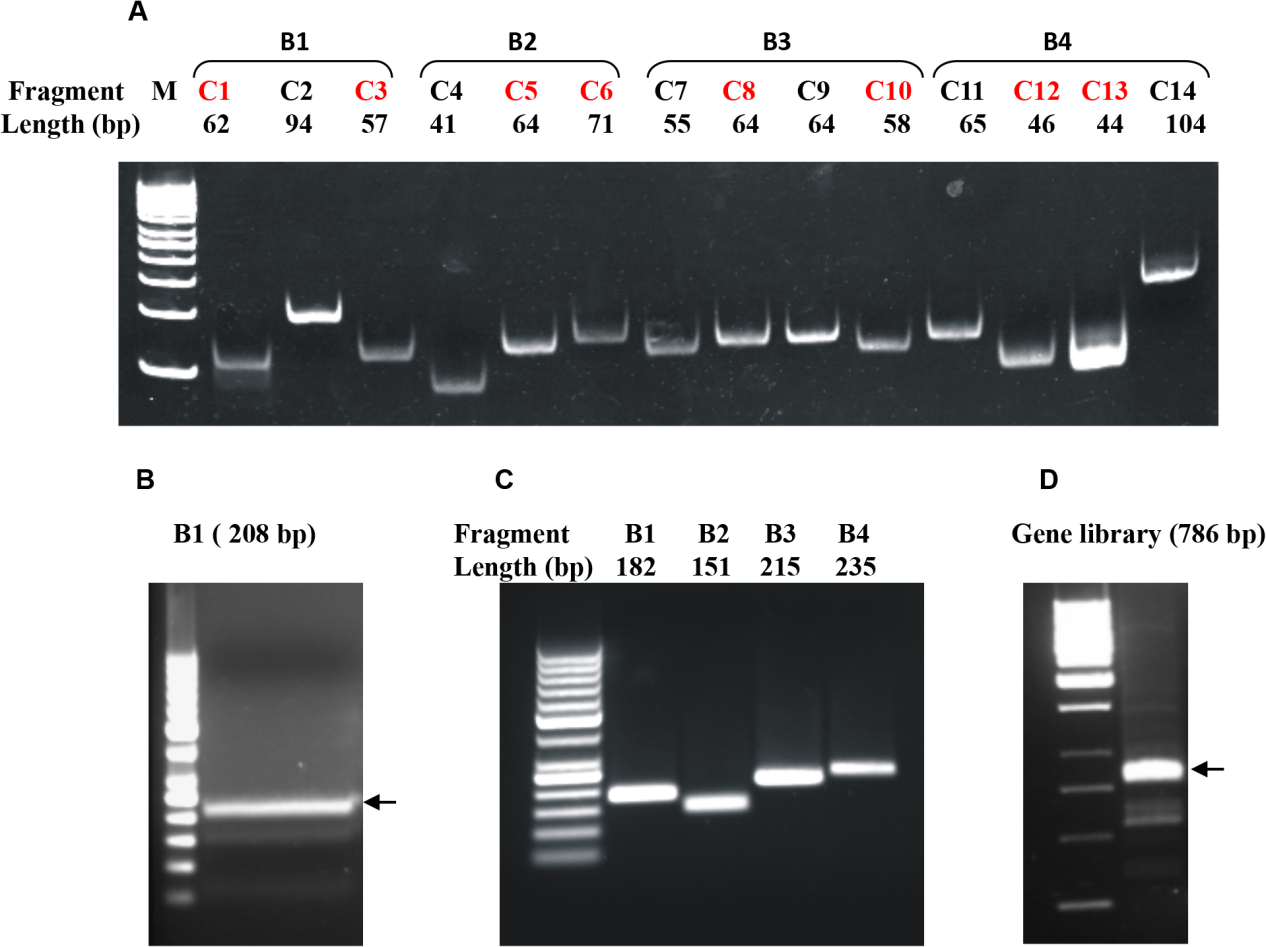
Assembly of the gene library. (A) Gel-eluted fragments from C-fragment libraries after restriction digestion (10% PAGE). The randomized fragments are marked in red; the brackets indicate the C-fragments, participating in one ligation assembly. Marker: 50 bp ladder (Thermo Scientific) (B) Ligation assembly of B1-fragment prior to gel elution. The full-length ligation product is indicated with an arrow. The length of the assembled B1 fragment with flanking BsaI restriction sites comprises 208 bp. Marker: 50 bp ladder (Thermo Scientific) (C) Gel-eluted fragments from B-fragment libraries. The length of the B1 fragment, liberated from the plasmid vector after restriction digestion comprises 182 bp. (D) Gene library ligation assembly. The full-length ligation product is indicated with an arrow. Marker: 1 kb ladder (Thermo Scientific).

In the next stage of hierarchical assembly, C-fragments were ligated to obtain B-fragments (compare overview in Fig 3A). The first step was liberation of C-fragments from their respective plasmids. For fragments located in the interior of a B-fragment, this was achieved simply by cleavage with Esp31. Fragments located at the edge of a B-fragment were isolated in three consecutive steps: *(i)* plasmid linearization with SmiI generating blunt ends, *(ii)* dephosphorylation, *(iii)* cleavage with Esp31. C-fragments isolated from plasmids were analyzed by PAGE (Fig 4A).

Fragments B1 to B4 were assembled by ligation of three or four C-fragments each (Fig 4B). Ligations were unequivocally directed by unique overlapping ends created by Esp31 (Table 1) and terminated by the unphosphorylated blunt ends of the ligation products. Assembled B-fragments were purified by preparative agarose gel electrophoresis (Fig 4C) and inserted into TOPO-blunt vector. Transformation efficiencies of the B-libraries were in the order of 10^4^ (Table 2) which is a factor of 10 higher than those of the C-libraries. This intermediate diversity theoretically allows the combinatorial assembly of 10^16^ unique genes, still orders of magnitude more than actually handled by any current gene library technology (compare above).

For biochemical reasons, fragment C5 was synthesized in two versions, one with two randomized positions and one without any. Hence, two different corresponding B-fragments were assembled, B2 containing six and B2* containing four randomized positions. A number of unselected clones from all four B-fragment libraries (with exception of B2*) were sequenced and statistically analyzed as described in "library analysis" (see below).

#### Assembly of full-length gene libraries

Full-length gene libraries were assembled by ligation of B-fragments in a similar way as B-fragments from C-fragments. Fragments B1, B2/B2*, B3 and B4 were liberated from their plasmid vectors by digestion with BsaI and separated from linear vector by preparative agarose gel electrophoresis. Fragments eluted from gel are shown in Fig 4C.

Sublibrary Fragments B1- B4 were assembled by ligation in a one-pot reaction, result is shown in Fig 4D. Unlike in the assembly of B-fragments, ligation was terminated not by unphosphorylated DNA termini but by incompatible protruding ends. Through incorporation of either B2 or B2* in the ligation mixture, two libraries were generated, L26 and L24. After elution from agarose gel, the full-length gene assembly products were directionally inserted into modified expression vector pASK-08 (see Materials and Methods). Experimental library diversity of L26 and L24 was 1.8 x 10^8^ and 1.2 x 10^6^ independent clones, respectively.

### Library analysis

As outlined above, chemical DNA synthesis and hierarchical module assembly were planned in such a way as to create two gene libraries, L26 and L24, harboring arbitrarily set features such as number and location of randomized codons, average number of codon exchanges per gene and frequency distribution of codons for different amino acids at randomized sites. To which degree these preplanned features are represented in the actual synthesis product can only be determined by DNA sequence analysis of a large number of unselected clones. At the same time, such an analysis yields information on the general fidelity of chemical DNA synthesis plus enzymatic assembly of synthetic DNA modules to gene-size, double-stranded DNA. We sequenced a total of 828 plasmid clones taken from all three hierarchical levels of library synthesis containing a total of 10,116 randomized codon positions (Table 3) and subjected the results to statistical evaluation.

**Table 3:** Observed average substitution rates.

	Number of triplets sequences	Average exchange rate
**Cumulative**	**10116**	**0.27**
**L**	7712	0.27
**B**	1552	0.27
**C**	852	0.31

Codon exchange rates per randomized position in C- and B-fragment libraries and in the final gene libraries (L = L24+L26).

#### Sequence Analysis

**Fig 5:**
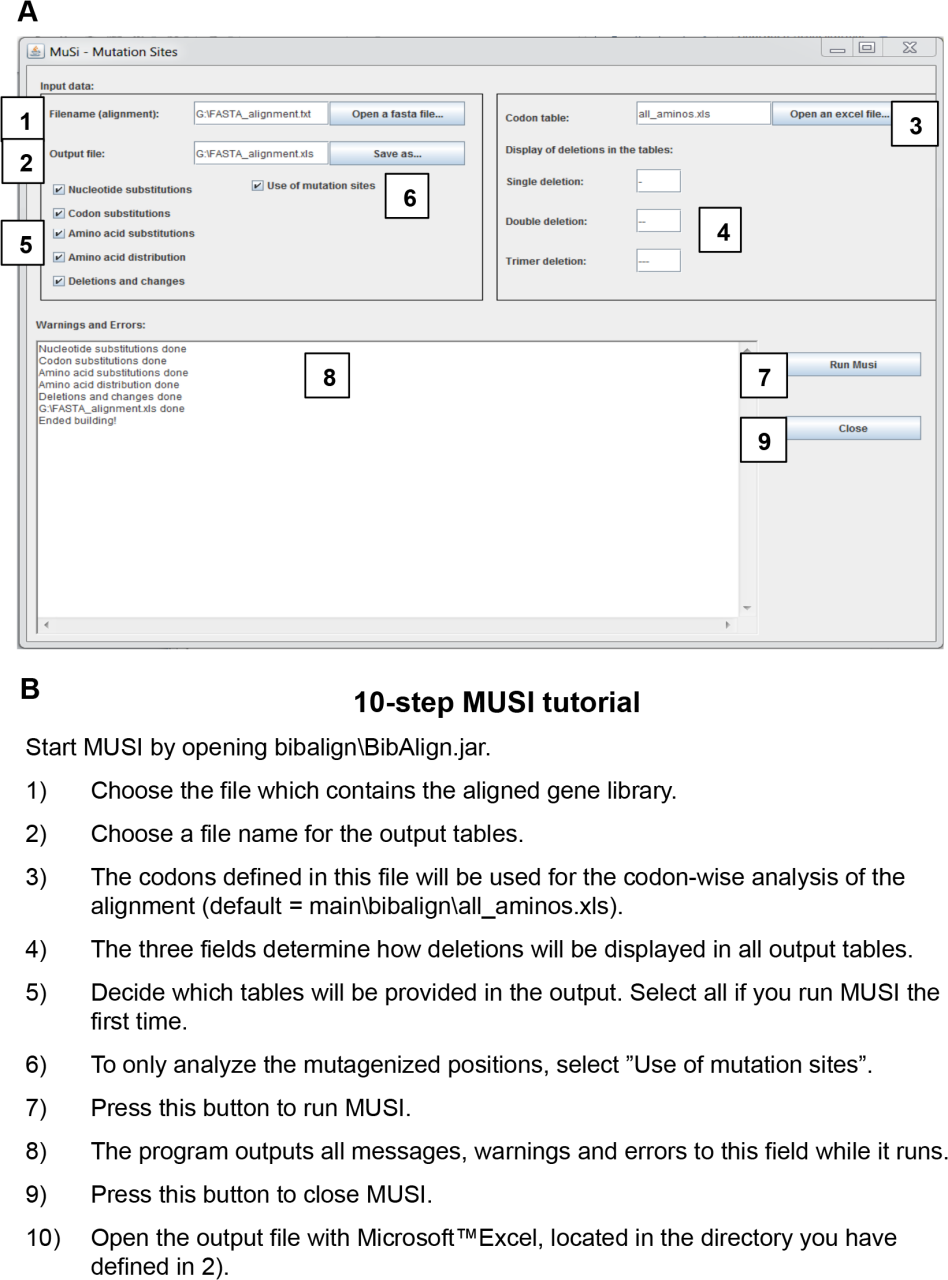
Overview of MUSI software. (A) Screen-shot of the MUSI software. (B) 10-step MUSI tutorial. For details, see Materials and methods and consult the manual (manual.txt), included in the download package.

Experimental DNA sequence analysis was carried out as described under Materials and Methods. In order to extract relevant information from crude primary data sets, software tool MUSI (Mutation Sites) was developed, which has been designed to be applicable to a broad range of mutagenesis experiments. MUSI software can be used for analysis of mutated gene sequences by site-directed mutagenesis and by standard methods of random mutagenesis like error-prone PCR. Starting from multiple sequence alignments, MUSI extracts sequence variations at randomized sites and throughout the entire gene length. It renders the results susceptible to further statistical analysis by exporting them into Excel tables. MUSI is freely available for download at http://bioinf.uni-greifswald.de/bioinf/musi/musi.html. A short workflow of MUSI is presented in Fig 5. For further details see Materials and Methods.

#### Overall Substitution Rates

As pointed out under *Library Design*, trinucleotide mixtures were adjusted such as to produce substitutions at any randomized position with a probability of 0.28. Table 3 summarizes substitution rates as observed by sequencing 828 clones of 14 different full size and fragment libraries. We define the observed substitution rate at a given randomized position as the number of non-wild-type codons found at that position, divided by the number of clones sequenced to determine that number. The close agreement of observed and expected substitution rate is a first indicator of good control over gene synthesis with partial randomization.

#### Individual Substitution Rates

Free choice of relative representation of different codons at randomized sites is an important element of controlled library synthesis. These representations are established by coupling reactions in which different trinucleotides are present in excess and compete in mixture for free 5'-hydroxyl groups of growing, immobilized chains. Since the elongation reactions are irreversible, the representations are controlled by the forward reaction rates. Assuming first-order reactions and equal rate constants, codon representations should reflect their molar ratios in the codon mixtures. Molar ratios can be corrected by empirical adjustment factors to compensate for different reactivities (compare below).

Again, DNA sequence analysis of a large number of clones must be used for assessing to what degree such synthesis plans were borne out by experiment. The problem can be looked at from two complementary sides: One can measure the rates by which individual resident codons are substituted by any of the eighteen other codons and one can measure the efficiencies of different trinucleotides to compete with others in replacing a resident codon. Both calculations were carried out on the cumulative data set stated in Table 3 (10,116 codons at randomized positions, 2,762 codon substitutions).

**Fig 6:**
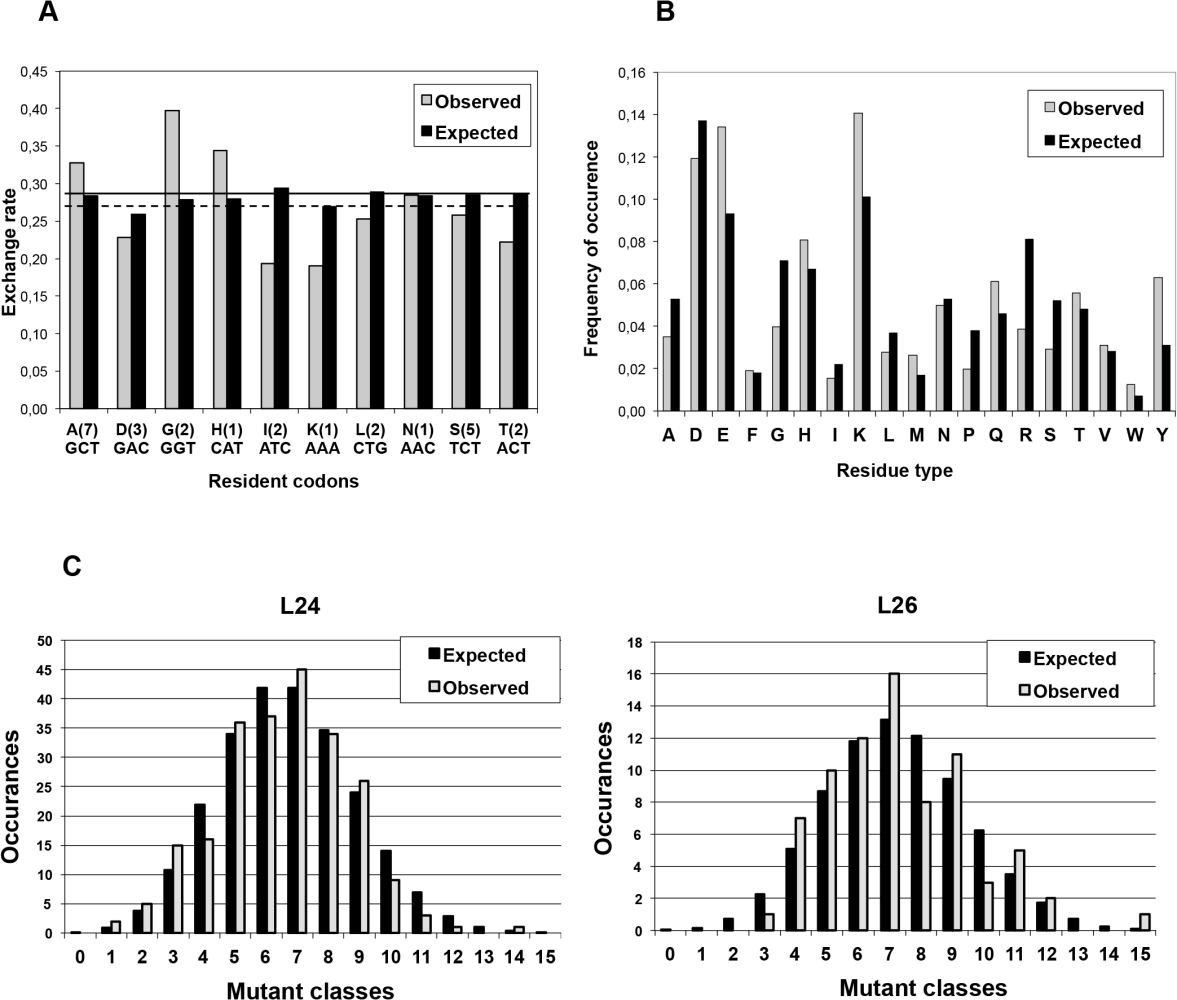
Library analysis. (A) Exchange rate per type of resident amino acid. Comparison of the expected exchange probabilities (the fraction of foreign trimers in each trinucleotide mixture) with the observed exchange rate per type of resident codon (cumulative, sample size: 2762 exchanges). The number of resident amino acids among the 26 selected positions is indicated in parentheses, below is the respective codon. The differences in the expected exchange probabilities per type of residue are due to the biased composition of the trimer mix. The thick horizontal line indicates the average exchange probability (0.28), the dashed line–the average observed exchange rate (0.27). (B) Pertaining to replacing residues. Comparison of the expected frequency distribution of the amino acid exchanges (the molar ratio of each trimer in the trinucleotide mixture) with the observed frequency distribution for a sample of 2762 trinucleotide exchanges. (C) Binomial distribution of the number of mutations in L24 and L26. The observed distribution of the number of mutations per molecule (mutant class) is compared with the expected one (number of representatives in each mutant class for the given sample size = occurrences). The expected distribution of the mutant classes (L24: k = 1–24; L26: k = 1–26) is calculated according to the binomial distribution for a sample size of 239 sequences (L24) and 76 sequences (L26).

The 26 codons of the *thisF* gene selected for controlled partial randomization comprise ten different wild-type ("resident") codons, occurring between one and seven times. Their rates of substitution for non-resident codons are displayed in Fig 6A.

There is fairly good agreement between expected and observed substitution rates (due to different contributions made to the standard mixture by different trinucleotides, calculated rates are not identical throughout). Small but significant deviations are, however, apparent: Exchange rates observed at Gly and His codons are markedly higher, those at Ile, Lys and Thr codons lower than expected. This could be due to either inaccurate weighing in the preparation of codon mixtures or to intrinsic differences in coupling efficiencies, which were not compensated appropriately by individual adjustment factors (compare above). In the latter case, the observed over- and underrepresentations should be mirrored by opposite effects in the set of rates with which different codons recruited from the standard mixture substitute resident codons; relevant data are illustrated in Fig 6B.

With respect to this latter question, no compelling message emerges from Fig 6B: In some cases the agreement between expected and observed representation is good, in others it is not–the most drastic deviations being seen with Tyr (two-fold over-represented) and Arg (two-fold under-represented). The mirror trend of over- and under-representations between Fig 6A and 6B, considered in the previous paragraph, is seen with Gly, Lys and Thr, but not with His and Ile, which would argue more in favour of weighing inaccuracies. It should, however, be kept in mind that in some cases *(i*.*e*. broken down to individual amino acids) the sample sizes on which Fig 6B is based are fairly small (0.01 normalized frequency of occurrence corresponds to not more than 28 cases).

#### Library Structure

The deliberately set substitution probability of 0.28 per randomized codon position is expected to result in an average number of 7.3 substitutions per gene in L26 and 6.7 substitutions in L24 with binomial distribution around these mean values. Fig 6C shows the experimentally determined distribution of both full size libraries–in comparison with the respective expectation. There is generally good agreement between observed and expected distribution and with L24 there is less fluctuation around expected values than with L26. This is not unexpected in view of the sample size of 239 sequences investigated for L24, compared to 76 sequences for L26.

#### Accuracy of Synthesis

All sequenced genes were correct ligation products of properly assembled fragments B1 to B4. Unintended deviations from wild type sequence, with the exception of very rare insertions are compiled in Table 4. Calculated per unit DNA length, single nucleotide substitutions and deletions of the Δ1 and Δ2 type are moderately higher with trinucleotides than with mononucleotides (2 to 4 times).

**Table 4:** Unplanned sequences.

	At randomized sites (10116 triplets)		Outside randomized sites (222564 nucleotides)	
	number	Rate	number	rate
**nucleotide substitutions** *	50	4.9.10^−3^	95	4.3.10^−4^
**Δ1**	51	5.10^−3^	189	8.4.10^−4^
**Δ2**	4	4.10^−4^	10	4.5.10^−5^
**Δ3**	181	1.8.10^−2^	4	1.8.10^−5^

Number and rate of occurrence of unplanned sequences at and outside the randomized sites.

* codons not in the trinucleotide synthetic mixture. Δ1: single nucleotide deletion; Δ2: double nucleotide deletion; Δ3: triple nucleotide deletion.

Deletions of three consecutive nucleotides (Δ3) associated with randomized positions occur in the percent range and are thus the most frequent of all types of unintended sequence deviations. This obviously reflects the unfavorable chain elongation kinetics of the trinucleotide synthons used (note that a missing trinucleotide at a randomized position requires failure of both the corresponding elongation reaction and the subsequent capping of the unreacted 5'-hydroxyl group by acetic anhydride).

While the occasional missing of a single codon at a randomized site may be a rather innocuous mistake, its frequency could be decreased by any of the following measures: *(i)* use of more reactive trinucleotide blocks, *(ii)* further increase in trinucleotide excess, *(iii)* further increase in reaction time, *(iv)* stronger capping conditions. With option *(iii)* one may run into the problem of partial detritylation of trimer building blocks due to their longer exposure to the mildly acidic coupling agent. In this context it is significant to note that only one instance was observed in which two trimers were incorporated in one step.

A total of 51 deletions of a single nucleotide (Δ1) were detected within the sample of 10116 sequenced randomized sites (Table 4). This corresponds to a 0.5% contamination of trinucleotides by dinucleotides (under the simplifying assumption of identical incorporation kinetics). 38 cases can be attributed to the incorporation of a dinucleotide of the standard set lacking its 5'-terminal residue, 2 to a lacking central residue, 1 to a lacking 3'-terminal residue and 10 cannot be explained as being derived from the trinucleotides of the standard set by lacking one residue. Taken together, these results point to failure of the last coupling step in a 3'→5' trinucleotide synthesis scheme as the most frequent source of contaminating dinucleotides.

#### Practical Diversity of Library

Only genes encoding full-length proteins with sequences within the constraints set by the synthetic design can count as contributing to library diversity in a practically useful way. If one includes in this count (the low number of) unintended nucleotide substitutions and Δ3 deletions, one arrives, in first approximation, at a practical library diversity by subtracting from the number of independent transformants the number of candidate clones carrying stop codons (not observed here) or frameshift mutations. With a gene length of 260 codons (Fig 1) and a cumulative frequency of Δ1 and Δ2 mutations per codon of 0.54% for randomized and 0.26% for non-randomized sites, the probability (P) for the occurrence of a frameshift-free gene for L24 is:

$$\text{P}={\left(1-0.0026\right)}^{236}\;*\;{\left(1–0.0054\right)}^{24}=0.5.$$

In other words: With the quality of reagents and the chemical procedures available for this study, half of all primary transformants are wasted as frameshift mutants. In comparison, other sources of diversity reduction, such as multiple occurrences of individual molecular species in the classes of very low number of mutations, are insignificant. The result highlights the tight quality constraints in chemical synthesis of gene libraries and points to limitations, which will need to be addressed first in any future efforts of further improvements.

#### Calculation of the actual library diversity

The true measure of library diversity, i.e. the functional library size, is the number of different molecular species, existing in the library population, corrected with the fraction of correctly assembled clones without frameshifts or stop codons. Standard methods for codon randomization produce redundant sequences and stop codons and the actual library diversity can be orders of magnitude below the reported number of transformants.

Here the actual diversity of the two libraries L24 and L26 is calculated, which represents the number of unique members that exist in any copy number, counting each single member only once. The calculation is based on the experimentally determined binomial distribution and assuming equal probability of occurrence for all possible clones.

The copy number of each molecular species *Ci*:

$$C_{i}=F_{i}∙L$$

$$F_{i}=\frac{P_{k,n,p}}{N_{i}}{}_{}$$

$$N_{i}=\left(\begin{array}{c} n\\ k \end{array}\right)18^{k}$$

where:

*F*_*i*_—fraction of one molecular species in the repertoire

*P*_*k*,*n*,*p*_−binomial distribution

*N*_*i*_—number of the molecular species in each mutant class

*n*–number of randomized positions (n = 24/26)

*k–*number of mutations in one mutant class (k = 1, 2, 3, …, 24, 25, 26)

18—number of the foreign amino acids

*L*–library size (number of independent clones)

The number of different molecular species (*S*) is calculated with the formula:

$$S=\sum \frac{L_{i}}{C_{i}}$$

The number of library members in each mutant class *(L*_*i*_ *)* is:

$$L_{i}=P_{k,n,p}∙L$$

where *C*_*i*_ is equal to *L*_*i*_ by *C*_*i*_< 1.

The calculated actual diversity after correction with a factor of 0.5 for frameshift-free genes represents 0.89 x 10^8^ and 0.6 x 10^6^ for L26 and L24 respectively.

For comparison, standard method for generation of gene libraries is the randomization with NNG/C codons, where N represents any nucleotide. This results in 32^n^ genes, coding for 20^n^ proteins, where n is the number of randomized positions. When increasing number of codons are randomized, there is a reduction in the efficiency of randomization due to inevitable cloning of redundant codons. For n = 26 the ratio genes: proteins is 2 x 10^5^. In contrast, the L26 library generated with the current method for combinatorial chemical synthesis results in 0.89 x 10^8^ unique proteins encoded by 0.9 x 10^8^ frameshift-free genes, thus the ratio genes: proteins is 1.01.

#### Fraction of conformationally stable proteins

**Fig 7:**
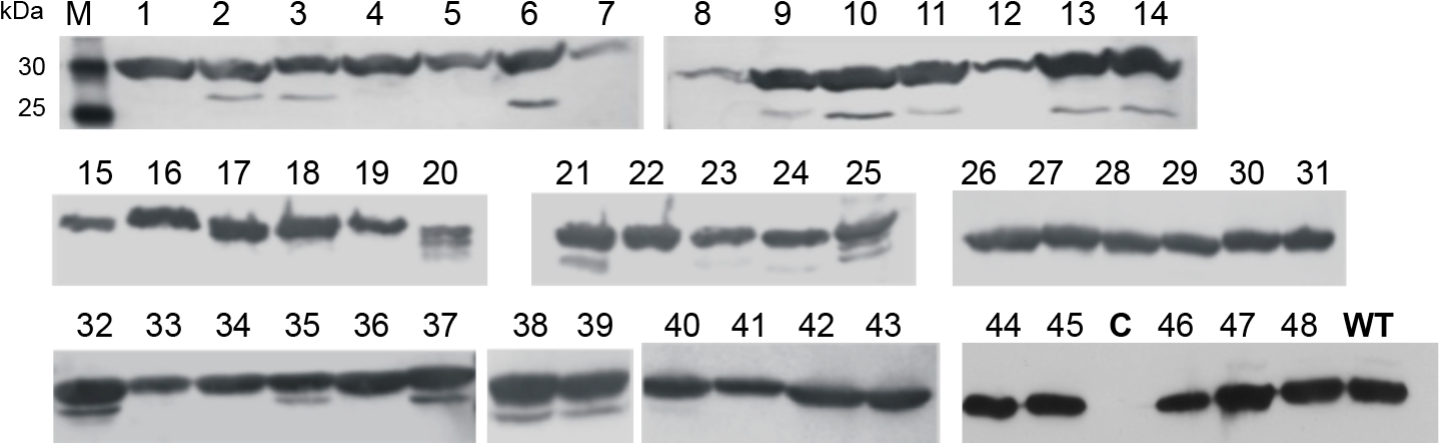
Proteins produced from gene variants chosen at random from library L24. Soluble crude protein extracts of *E*.*coli* DH5α after 4 h gene expression were analysed by Western blotting using *anti-*His-tag antibody. C- negative control: cells, transformed with empty vector; WT: wild type *thisF (*MW_tHisF-His6_ = 29 kDa); M–marker. Mutations in each clone are listed in S1 Table.

A sample of 48 clones was chosen at random from the subset of L24 genes having complete open reading frames (deletions of three consecutive nucleotides permitted). Levels of accumulation of protein in the soluble fraction of induced cells were visualized by subjecting cell lysates to SDS gel electrophoresis and Western blotting as shown in Fig 7. Robust protein accumulation is observed in the majority of cases, exceptions being clones #7, #8, #12, #20 and #25 which accumulate significantly less full-length protein and/or show multiple degradation bands. There is published evidence that within a set of sequence derivatives of one particular protein, solubility and (relative) protease resistance are good proxies for conformational stability (see, for example [41]). By that criterion, we estimate the fraction of conformationally stable proteins in the full-reading-frame subset of L24 to be ca. 90%.

The DNA sequence of all 48 clones was determined. Detailed results are given in Supplementary Material, S1 Table. The strong accumulators carried 6.0 replacements and 0.2 three-nucleotide deletions on average, the weak accumulators 8.4 replacements and 0.6 3N-deletions (note that in the latter case the sample comprises only five genes). The mean values for the complete sample are 6.3 and 0.25, respectively. The high proportion of stable proteins (90%), together with the asymmetric distribution of numbers of replacements between the two classes can be taken as indicating that the starting assumption of a set of amino acid residues with no influence on folding as well as the chosen randomization parameters are, in first approximation, justified.

#### tHisF Variants with HisF function

In a first test experiment for the feasibility of our method, we searched for clones that are able to complement an *E*. *coli* Δ*hisF* mutant. Wild type tHisF is known to complement histidine auxotrophic *E*. *coli* cells lacking a functional hisF gene [42]. We preferred this approach over a screen for new enzymatic functions, since we could make solid predictions about the number of clones we have to find, allowing us to test the method much more rigorously. Searching for new enzymatic activity and failing to detect them would not give a clear answer. That is, it would be unclear whether we just failed to detect those, whether there was intrinsic shortcoming in our procedure or whether the new activity could not be generated.

As shown above, library L24 contains ca. 50% complete open reading frames among which about 90% code for proteins accumulating in *E*. *coli* cells in soluble form. The frequency of the wild-type HisF sequence in the protein library corresponds to the Zero-term of the binomial distribution (about 1.4 x 10–4 for L26 –corrected for complete frames and fraction of stable proteins). Sampling 106 transformants in a routine experiment should therefore yield more than 100 wild type *hisF* genes. Moreover, one would expect an unknown number of mutants closely clustered around the wild type HisF in the sequence space and carrying amino acid exchanges specifically in the subset of the 26 randomized amino acid positions that are not essential for the HisF function. Table 5 gives a summary of the outcome of the experiment. Twelve protein variants were selected, among them the wild type HisF, as expected. This demonstrates that the approach is in principle viable. In sequenced clones, only nine of 26 randomized positions could be demonstrated to accept mutations under conservation of HisF function. The average number of amino acid substitutions in the selected set is 1.6. With respect to chemical characteristics of the residues involved, the observed substitutions are generally of a conservative nature. Taken together, rather stringent structural constraints emerge for HisF function. This result is not too surprising in view of the demanding two-step reaction catalyzed by HisF [42].

**Table 5:** Summary of genetic complementation results.

Clone	A8	A54	S55	H84	A128	A204	L222	A223	S225	Doubling time (min.)	Standard dev. (min.)
**WT**	** **									152	8
**F1**				R	G				T	126	5
**F2**	** **			R						147	11
**F3**				N					T	150	4
**F4**	** **	G	N							157	4
**F5**								T		160	10
**F6**	G									161	6
**F7**				Δ3					T	161	2
**F8**				N						162	9
**F9**				Δ3		V			T	172	11
**F10**							T			206	6
**F11**									T	143	2

Amino acid substitutions in library members that complemented the HisF deficiency. The growth rate of *ΔhisF* cells, transformed with the corresponding plasmid variants was determined. The doubling time is a mean of five independent experiments.

Since the diversity of the chemically produced library used for transformation is very much larger than 106, each candidate clone can be expected to code for a protein of unique sequence. This should make it possible to find candidates meeting certain arbitrarily set functional requirements as long as these are not too demanding with respect to protein structure. The high functional diversity of the library should significantly improve the outcome of future experiments for selection of catalytically performing proteins.

As already elaborated in the introduction, the claim that trinucleotides are superior in library construction to other methods for generating randomized oligonucleotides coding for protein variant could largely be demonstrated, although, as already discussed above, it might be improved by optimizing chemical synthesis in order to eliminate frameshift mutations. Another aspect is the introduction of the library into the cells. Here we used total gene synthesis with subsequent introduction of the gene variants into cells *via* transformation. An option to be considered is multiplex automated genome engineering (MAGE) described by the Church lab [43]. Here, a culture of *E*. *coli* cells is repeatedly transfected with the randomized oligonucleotides and the mutations are transferred to a gene residing either in the chromosome or on a plasmid with the help of the lambda red recombination system. This is certainly a viable option since this procedure can be repeated over and over, which might be advantageous for optimization of selected protein variants with low activity. The MAGE method enables generation of high sequence diversity. However, one loses some control of the degree of randomization if mutating multiple positions in a genome, which our method offers. Furthermore, there is no immediate estimate of the diversity of the *E*. *coli* population. The advantages of MAGE are mainly offered if the selection or screening procedure can be applied to proliferating *E*. *coli* cells. If, however, any other labor intensive screening procedure is necessary, it would be rather advantageous to keep complete control on the experimental parameters, in particular to know exactly the diversity of the library.

## Conclusions

**Fig 8:**
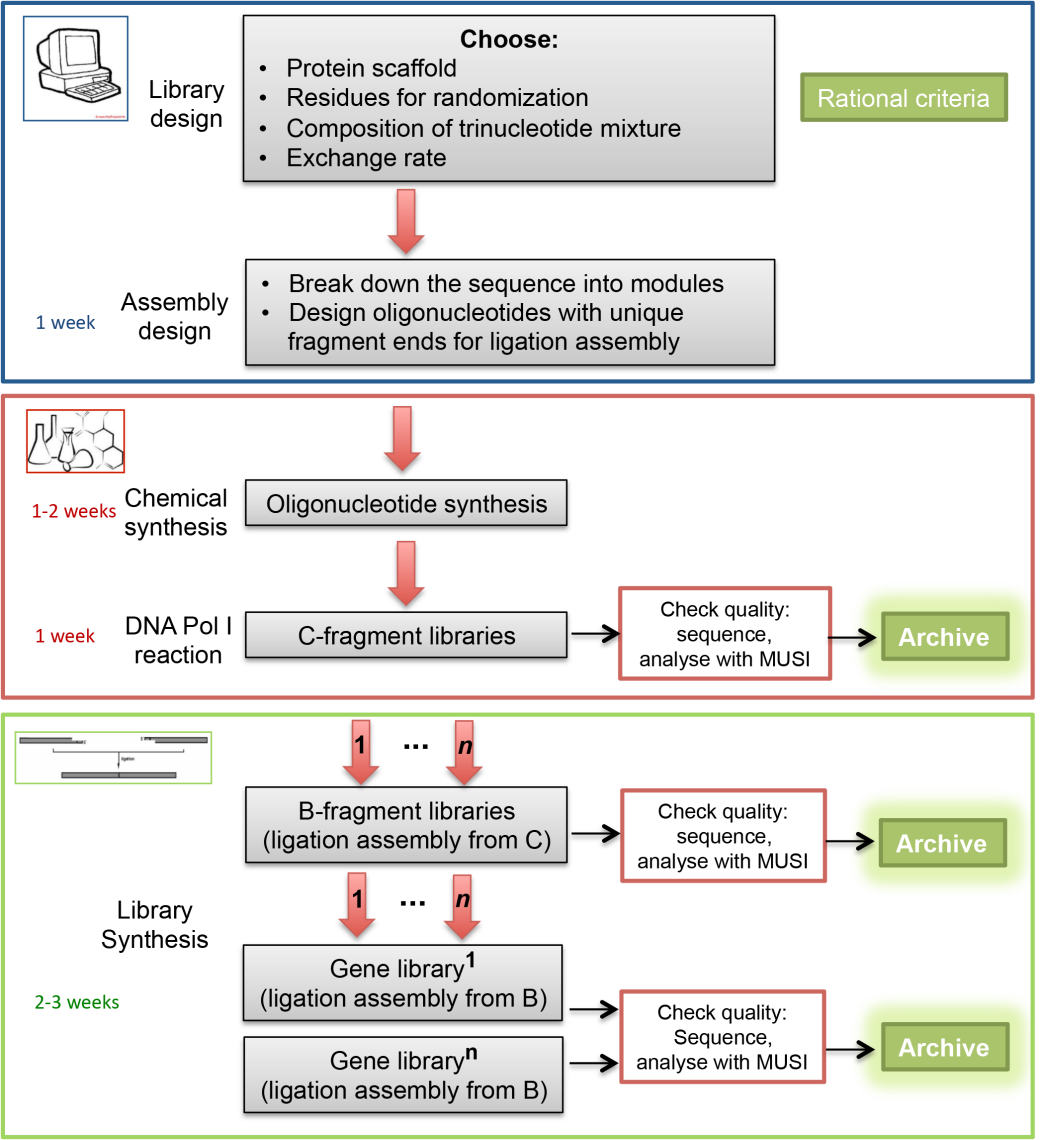
Summary workflow diagram of the method for combinatorial gene synthesis. *Library design*: the method imposes no restrictions to the number of residues selected for mutagenesis or to their location. The composition of the trinucleotide mixture and the exchange rate can be freely chosen without any limitations. Contact the oligonucleotide synthesis company of your choice well in advance and coordinate the purchase of trimer phosphoramidites. *Assembly design*: The fractionation of the gene sequence into modules depends on the locations of the residues for randomization. Divide the gene sequence into modules with a length between 40 bp and 90 bp, containing the mutagenized codons. The fragment borders of the modules should create unique overhands for ligation assembly after restriction digestion (see Table 1 and main text for details). Use PCR for longer stretches of wild type gene sequence. *Chemical synthesis*: Invest efforts and resources into high quality oligonucleotide synthesis as well as cloning and analysis of the C-libraries. The chemically synthesized diversity is stored in them and they are the starting point for all future gene libraries. Clone a wild type sequence, corresponding to each mutagenized module. *Library synthesis*: Generate B-fragment libraries by ligation from C-fragment libraries. Different B-fragment libraries (1, …, n) can be obtained in parallel by exchanging or mixing mutagenized modules with the corresponding wild type modules. Archive the B-libraries. Generate gene libraries by ligation assembly of B-libraries. Use the combinatorics approach for generation of multiple unique gene libraries.

The thorough statistical analysis demonstrates that trinucleotide randomization is a method of choice that allows any required subset of amino acids to be encoded exclusively and reliably. The use of trinucleotides mixtures for controlled randomization avoids the codon redundancy and the inevitable incorporation of stop codons when standard methods for randomization with degenerate oligonucleotides are used and achieves high functional diversity. No PCR was involved for amplification of mutagenized fragments and hence no concomitant skewing of library composition due to amplification bias. The new approach for combinatorial assembly of gene fragments combines the advantages of trimer phosphoramidites with easy and cost-effective design of a gene library. The cost of the gene library is generally restricted to the purchase of trimer phosphoramidites and oligonucleotide synthesis and depends on the number of positions for randomization. The method requires low cost consumables for the additional biochemical procedures and no special laboratory equipment. For a summary of the method, see Fig 8.

The splitting of the gene into multiple modules depends on the location and number of the randomized positions. There are no constrains as to the number of modules or the number of randomized codons per module. The length of the randomized modules is restricted by the synthesis of oligonucleotides up to a length of about 90 nucleotides. Longer stretches of gene sequence without randomized positions can be amplified by PCR. The fragment borders of the modules should create unique overhands for ligation assembly after restriction digestion. Since the restriction enzymes of Type IIS cleave an arbitrary sequence outside of their recognition sequence, the splitting of the gene into modules can be done at any nucleotide outside of the randomized positions.

The mutual exchange of a mutagenized library fragment with a wild type fragment or vice versa allows numerable iterations at a later stage without the need to start “from the beginning”. The mutation rate at specific positions can be controlled by simply mixing or exchanging the wild type and the randomized fragment without need of new chemical synthesis, which considerably reduces the costs for library synthesis. The easy control over the combinatorics and the reliable storage of low diversity at the stage of fragment libraries allows the generation of new unique libraries by each ligation and transformation procedure. The new method for combinatorial gene synthesis can be broadly applied to engineering of gene libraries for screening of new functional mutants, being directly relevant for industries such as biomedicine or biotechnology.

## Supporting Information

S1 Fig PAGE analysis of Klenow fill-in products. (PDF)

S1 Table Amino acid substitutions in 48 clones, examined for accumulation of soluble HisF protein. (PDF)
