# Supplementary material for: A Robust and Versatile Method of Combinatorial Chemical Synthesis of Gene Libraries via Hierarchical Assembly of Partially Randomized Modules
Source: PLoS One. 2015 Sep 10;10(9):e0136778. doi: 10.1371/journal.pone.0136778 (PMC4565649; doi:10.1371/journal.pone.0136778)
Supplement: S1 Fig — (PDF) [file pone.0136778.s001.pdf]

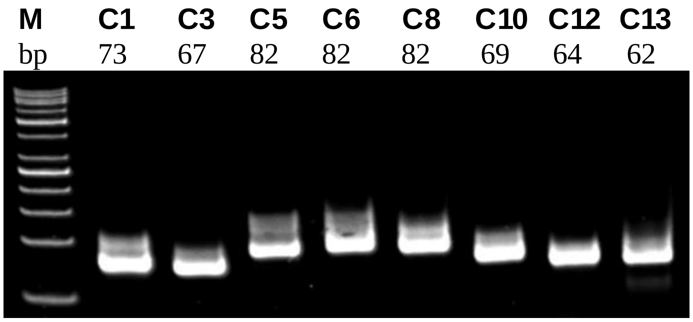

S1 Fig. PAGE analysis of Klenow fill-in products. 15 pmol of each randomized double stranded fragment were applied on a 10% PAGE for evaluation of the integrity of the elongation products; M=50 bp ladder (Invitrogen).
