# Supplementary material for: A Robust and Versatile Method of Combinatorial Chemical Synthesis of Gene Libraries via Hierarchical Assembly of Partially Randomized Modules
Source: PLoS One. 2015 Sep 10;10(9):e0136778. doi: 10.1371/journal.pone.0136778 (PMC4565649; doi:10.1371/journal.pone.0136778)
Supplement: S1 Table — (PDF) [file pone.0136778.s002.pdf]

S1 Table. Amino acid substitutions in 48 clones, examined for accumulation of soluble HisF protein.

High level of accumulation of soluble protein

Clone	Substitu-tions	Δ3	A 8	D 11	L 50	I 52	A 54	*S 55*	S 101	*N 103*	T 104	A 105	A 128	D 130	S 144	T 171	I 173	D 176	*G 177*	K 179	S 201	A 204	L 222	A 223	A 224	S 225
**1**	1	1																			Δ3		K			
**2**	8	0			G		Y	D					E		Q		Y		S							T
**3[a]**	6	2						K						F	Δ3		E		H		Y			T	Δ3	
**4**	8	0	K				L			D	K						F				K	S			K	
**5[b]**	8	0			T		E	D								D	G	E			P			K		
**6**	5	1					H	R										K	D						Δ3	N
**9**	4	0	D																		A	H				I
**10**	4	0					H											A				N		I		
**11**	4	0						Q						V				G					E			
**13**	8	0						K		D	K			S	K						Y			T	D	
**14**	8	0	K	Q			G	N											Q			H	T		M	
**15**	8	0						Q					K					I	L		Y	H		T		K
**16**	8	0						T	F				E	K			D		R			T		K		
**17[c]**	4	0									W			E				A							K	
**18**	5	0									W								V	E		K		T		
**19**	9	0		V		A	G			D	K					E	Y				H				K	
**21**	5	0	M	H			D										D									D
**22**	6	0			P					K					T		E	E							T	
**23**	9	0					E	H		D	K								D			M		M	H	K
**24**	3	0						K						E										K		
**26**	5	0						Q								Y				T			Q			G
**27**	10	0	N		M		N	E		K				R		Y				T			D			D
**28**	7	0											E			N		G		D	Q		N		K	
**29[d]**	5	0				D	H										Q							K	M	
**30**	7	0					K			H	R						E		E	H						I
**31**	5	0						K			P										V	H		K		
**32**	2	0					Q	E																		
**33**	3	1						F		E					Δ3							Y				
**34**	10	0		N		A	G			D	K				N	K	A						Q			I
**35**	6	0					D						E	R					V					Y		K
**36**	6	1	F	E											Δ3			Y					E	E		G
**37**	9	0	Y				Q	E					E	A		S			Q					E	N	
**38**	7	0					K								R	D	V					H		M	L	
**39**	5	0	H				L										F					H				I
**40**	5	0		S			K									L						R				T
**41**	8	0		R						D	K						E	E			Y			K	H	
**42**	4	1						Δ3					D								A				N	N
**43e**	9	0					Y	G		L	G						Y		E	F				K	K	
**44**	7	0					V			D	K										N	H	D		N	
**45**	5	1					L			K					Δ3						Y	E			T	
**46**	3	1	V	Δ3			D																		R	
**47**	6	0		Y												E					E		T	T		T
**48**	9	0		S	Q									A	Q			S	Y		K	H	E			

Low level of accumulation of soluble protein

Clone	Substitu-tions	Δ3	A 8	D 11	L 50	I 52	A 54	S 55	S 101	N 103	T 104	A 105	A 128	D 130	S 144	T 171	I 173	D 176	G 177	K 179	S 201	A 204	L 222	A 223	A 224	S 225
**7**	7	1	V				D	F		Y		D						E	Δ3				D			
**8**	9	1	Δ3		E	K		E					K		V				K		H			K	M	
**12**[f]	7	0			T								E	R					Y		R	H		K		
**20**	10	1	D	Δ3	M		N	E			W								Q			Y	D	H	E	
**25**	9	0			M		Q	E							G		N	I	D		K			K		

a: GTT/ATT (V56I); b: GAG/GTG (E57V); c: AAG/ATG (K13M); d: GAG/AAG (E161K), GGA/AGA (G166R); e: GTT/ATT (V56I), GCT-TCT (A218S); f: GCG/GGG (A221G)
